# Impact of Early Chest Radiography on Delay in Pulmonary Tuberculosis Case Notification in Ethiopia

**DOI:** 10.4103/ijmy.ijmy_216_21

**Published:** 2021

**Authors:** Hussen Mohammed, Lemessa Oljira, Kedir Teji Roba, Esther Ngadaya, Dagmawit Tesfaye, Tsegahun Manyazewal, Getnet Yimer

**Affiliations:** 1 Department of Public Health, College of Medicine and Health Sciences, Dire Dawa University, Dire Dawa,; 2 Centre for Innovative Drug Development and Therapeutic Trials for Africa, College of Health Sciences, Addis Ababa University,; 3 Department of Public Health, School of Public Health, College of Health and Medical Sciences, Haramaya University,; 4 Department of Nursing, School of Nursing and Midwifery, College of Health and Medical Sciences, Haramaya University, Harar, Ethiopia,; 5 Muhimbili Research Centre, National Institute for Medical Research, Dares Saalem, Tanzania; 6 Ohio State Global One Health Initiative, Office of International Affairs, The Ohio State University, Addis Ababa,

**Keywords:** Active case-finding, chest radiography, delay, diagnosis, Ethiopia, screening, tuberculosis, Xpert mycobacterium tuberculosis/RIF assay, X-ray

## Abstract

**Background:**

One-third of tuberculosis (TB) cases are missed each year and delays in the diagnosis of TB are hampering the whole cascade of care. Early chest X-ray (CXR) in patients with cough irrespective of duration may reduce TB diagnostic and treatment delays and increase the number of TB patients put into TB care. We aimed to evaluate the impact of CXR on delay in the diagnosis of pulmonary tuberculosis (PTB) among people with cough of any duration.

**Methods:**

A facility-based cross-sectional study was conducted in four selected health facilities from two regions and two city administrations of Ethiopia. Patients who sought health care were screened for cough of any duration, and those with cough underwent CXR for PTB and their sputum specimens were tested for microbiological confirmation. Delays were followed up and calculated using median and inter-quartile range (IQR) to summarize (first onset of cough to first facility visit, ≥15 days), diagnosis delay (first facility visit to date of PTB diagnosis, >7 days), and total delay (first onset of cough to date of PTB diagnosis, >21 days). Kruskal–Wallis and Mann–Witney tests were used to compare the delays among independent variables.

**Results:**

A total of 309 PTB cases were consecutively diagnosed of 1853 presumptive TB cases recruited in the study that were identified from 2647 people who reported cough of any duration. The median (IQR) of patient delay, diagnosis delay, and the total delay was 30 (16–44), 1 (0–3), and 31 (19–48) days, respectively. Patients’ delay contributed a great role in the total delay, 201/209 (96.2%). Median diagnosis delay was higher among those that visited health center, diagnosed at a facility that had no Xpert mycobacterium tuberculosis (MTB)/RIF assay, radiologist, or CXR (*P* < 0.05). Factors associated with patients delay were history of previous TB treatment (adjusted prevalence ratio [aPR] = 0.79, 95% confidence interval [CI]: 0.63–0.99) and history of weight loss (aPR = 1.12; 95% CI: 1.0–1.25). Early CXR screening for cough of <2 weeks duration significantly reduced the patients’ delay and thus the total delay, but not diagnostic delay alone.

**Conclusion:**

Early screening using CXR minimized delays in the diagnosis of PTB among people with cough of any duration. Patients’ delay was largest and contributed great role in the delay of TB cases. Screening by cough of any duration and/or CXR among people seeking healthcare along with ensuring the availability of Xpert MTB/RIF assay and skilled human power at primary healthcare facilities are important to reduce patient and diagnostic delays of PTB in Ethiopia.

## Introduction

One-third of tuberculosis (TB) cases are missed each year and delays in the diagnosis of TB are hampering the whole cascade of care.^[1]^ The emergence of severe forms of drug resistance and a slower decline of new cases revealed that expanding TB service coverage is not enough to end TB.^[2–4]^ In Ethiopia, the effectiveness of the national TB program (NTP) calls for an early case finding and treatment of TB and decentralize services to reach the communities at large;^[5]^ however, the program focuses on passive case-finding that potentially misses many cases to diagnose.^[6]^ A third of TB cases have been missed in Ethiopia^[7]^ and a couple of undiagnosed TB cases are identified in the community that hiddenly transmit the disease.^[8]^

Realizing early TB case finding and treatment requires, individual’s healthcare-seeking behavior, access to quality diagnostics, and a vigilant healthcare workforce.^[9,10]^ However, previous studies in Ethiopia reported low healthcare-seeking behavior among presumptive TB cases,^[11]^ poor quality of TB diagnosis and care,^[12]^ and delayed diagnosis and treatment of TB.^[13]^ The use of chest X-ray (CXR) in reducing diagnostic delay in Ethiopia has not been studied well, while available evidence shows variation based on the availability of CXR and other microbiological tools in a facility.^[14]^

Delay in the diagnosis and treatment of TB cases has several consequences; it leads patients to serious morbidity and death, overburdens healthcare facilities to manage severe cases, and increases disease transmission in the community.^[15]^ The problem is higher in resource-constrained high burden countries as it has an association with the overall burden of TB in a given country and its capacity to early diagnose and treat cases.^[16]^

For the past 10 years, high burden countries have been implementing TB screening to fill in case detection gaps and reduce diagnostic delays,^[17]^ and Ethiopia has been in the loop. Delay in the diagnosis of TB endured obstacles in Ethiopia,^[5]^ and health facility-driven delays were frequent among smear-negative, first sought health care, and among rural.^[5,18,19]^ A previous study showed that many patients visited several health facilities up to four rounds with a median of two before getting their final TB diagnosis^[20]^ and there have been many challenges that contribute to the low case-finding status of the country.^[21,22]^ However, evidence and recommendations on how to mitigate the delays are limited. Evidence of how to controlling missed opportunities through active case finding and the early use of CXR is also limited. Some studies reported a reduction in diagnosis delay due to screening of everyone for TB regardless of the duration of cough.^[23,24]^

Therefore, this study aimed to evaluate the impact of CXR on delay in the diagnosis of pulmonary tuberculosis (PTB) among people with cough of any duration.

## Methods

### Study settings and participants

**Figure 1:**
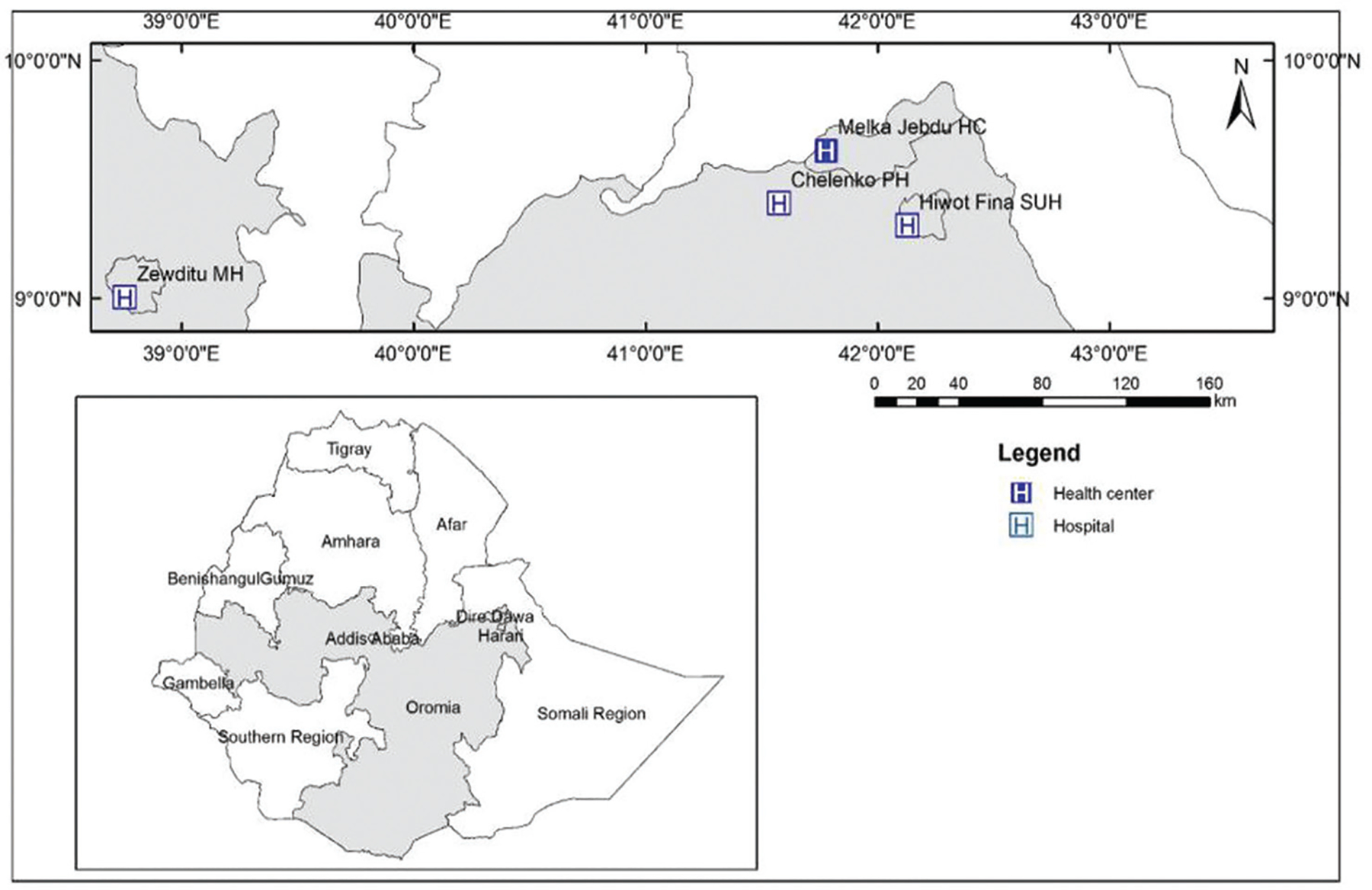
Map of study sites

Health facility-based cross-sectional study was performed in four health facilities that were selected from two national regional states (Oromia and Harari) and two city administrations (Addis Ababa and Dire Dawa) [Figure 1]. These facilities were randomly selected from respective stratified settings by urban and rural. In summary, Chelenko Primary Hospital was from Oromia National Regional State, Hiwot Fina Specialized University Hospital from Harari Regional State, and Zewditu Memorial Hospital and Melka Jebdu Health Center from Addis Ababa and Dire Dawa city administration, respectively. Hiwot Fina Specialized University Hospital and Zewditu Memorial Hospital were from an urban setting. Chelenko Primary Hospital and Melka Jebdu Health Center were from rural settings. A study was conducted from July 2019 to March 2020 and from August 2020 to December 2020. The study was interrupted because of COVID-19 pandemic from April 2020 to July, 2020.

Participants were patients who came to the health care facilities to sought health care at outpatients and those who visited the reproductive and child health units. The patients were screened for cough of any duration, and those with cough underwent CXR for PTB. For individuals such as pregnant women, diabetic patients, and anti-retroviral patients that could not undergo CXR, they were nonchest X-ray group and their screening followed that symptom screening for cough of any duration. Sputum specimens were tested for microbiological confirmation.

To calculate the sample size we made the following assumption: 95% confidence interval (CI), 5% margin of error, 72.3% proportion of total delay in nationwide study survey,^[5]^ using single population formula, we calculated 309 PTB cases and included all PTB cases diagnosed from people screened by the cough of any duration and/or CXR at health facilities.

Measurements of delays:

- Patient delay was defined for this study as the time between the first symptom (cough) to date of seeking health care, if ≥15 days, it was taken as patients’ delay^[5]^
- Diagnostic delay was the interval between the first visits to the health facility to the date of diagnosis for PTB. Time >7 days was considered indicative of delay
- Total delay was defined as the total time interval (days) between the onset of a symptom (cough) related to PTB to date of diagnosis and if >21 days, it was taken as total delay.^[5,18]^

### Data collection

A questionnaire was used to collect data by trained data collectors, controlling also recall biases.^[25]^ When patients visited study health facilities, they were screened and recruited immediately for those who fulfilled the eligibility criteria. The date of health facility visit, first dates of symptom (cough), and dates of diagnosis were captured using the study questionnaire. CXR and laboratory findings captured. The duration of delays for patients, diagnosis, and the total delay were calculated using these data.

### Chest X-ray and microbiological diagnosis

**Figure 2:**
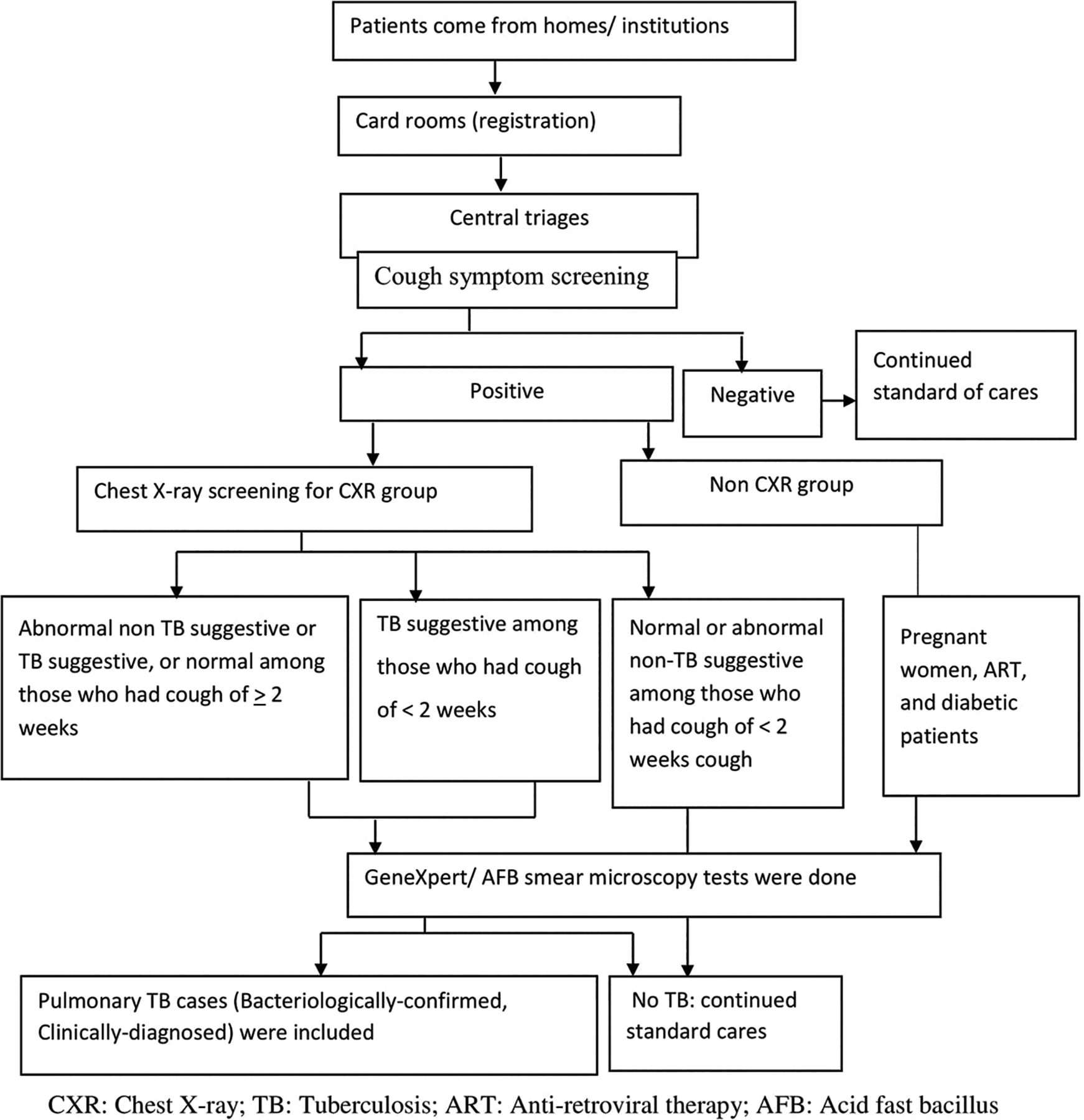
Screening by cough of any duration and/or CXR among people sought health care and diagnosis work flow

Chest X-ray was offered for the participants. The participants were furthermore stratified by cough duration (<2 weeks and ≥2 weeks). Sputum specimens were collected and microbiological confirmation was performed among those with CXR results of TB suggestive for <2 weeks cough duration and those with CXR results of all types (normal, TB suggestive, and non-TB suggestive) for ≥2 weeks cough duration. A strong emphasis was given to keep the quality of CXR reading and CXRs were read by radiologists. For health center that lack CXR machine, patients were transported to a nearby public healthcare facility with a CXR machine and the study covers the cost. Microbiological confirmation was as per the national TB guideline which was either Xpert mycobacterium tuberculosis (MTB)/RIF assay or acid-fast bacilli (AFB) microscopy^[7]^ [Figure 2].

Data collection was followed up daily to ensure data quality. The principal investigator verified the data reported on the questionnaires by reviewing study participants’ source documents for completeness and accuracy. Data were stored in key-locked metal cabinets to keep the security.

### Data analysis

Data was analyzed using Stata version 14.0 (StataCorp, College Station, Texas, USA). Data were summarized in frequencies and median depending on the type of study variables. Median and inter-quartile range (IQR) were used to summarize patient, diagnosis, and total delays among PTB cases. Nonparametric Mann–Whitney and Kruskal–Wallis tests were used for skewed delay data. Based on this, Mann–Whitney test was used to compare delays between two categories of an explanatory variable, and Kruskal–Wallis if a variable had three or more groups.^[26]^ Prevalence ratios were the estimates used with 95% CIs for the modified Poisson regression model,^[27]^ which was used to reveal factors associated with patient’s delay ≥15 days and total delay >21 days. Statistical significance was determined at *P* < 0.05.

## Results

### Socio-demographic characteristics

A total of 309 pulmonary tuberculosis (PTB) cases were consecutively diagnosed of 1853 presumptive TB cases recruited in the study that were identified from 2647 people who reported cough of any duration at the study health facilities. Of 309 PTB cases, 163 (52.7%) were males, 176 (57%) married, and 105 (34%) peasant in occupation. The median (IQR) age of TB cases was 27 (20–35) years and with a range from 1 to 80 years. A third of TB cases were in the age group of 25–34 years, 97 (31.4%). Those that never attended education constituted 147 (47.6%) [Table 1]. The majority of TB patients visited at public institutions, 284 (92%). About 1 in 10 PTB cases had a history of previous TB treatment [Table 2].

### Patient delay

The median (IQR) of patient delay was 30 (16–44) days, ranged from 1 day to 212 days. Of 298 PTB cases, those who visited health facilities to seek care for cough in recognized periods of two weeks were 57 (18.5%). The majority, 252 (81.6%) sought health care after ≥15 days. Median patient delays were higher among rural residents, those who visited hospitals, and were not treated for TB previously (Kruskal–Wallis or Mann–Whitney tests, *P* < 0.05) [Table 2]. Of all, 252 (81.6%) patients’ delay (sought health care after ≥15 days), in which 178/252 (70.6%) delayed for 30 days or more without seeking health care. More than three out of four, 189/252 (83.6%) were from rural.

### Diagnostic delay

Of all, 301 (97.4%), knew their diagnosis within 7 days (<7 days). The median (IQR) diagnosis delay was 1 (0–3) day, ranged 1–17 days. Following contact with a health care provider, notably, only 8 (2.6%) were diagnosed with diagnostic delay after 7 days (1 week). Zero-day was to mean same day, the difference between visit date and diagnosis completed date, among 86 (27.8%) diagnosis was completed on the same day. Median diagnosis delays were higher among those diagnosed at the facilities that were found at rural, those visited health centers, those diagnosed at the facility that had no Xpert, had no radiologist, or had no CXR (Kruskal–Wallis or Mann–Whitney tests, *P* < 0.05) [Table 3].

The diagnostic delay was varied by health facilities and highest at the health center, 3 (2–4) days that had only smear microscopy. Delays were different by screening algorithms that diagnostic delay was higher among those who screened by symptom followed by chest X-ray (CXR) with cough <2 weeks duration compared with CXR group with cough ≥2 weeks or screened by symptom only but it reduced the patients’ delay significantly [Table 4].

### Total delay

The median (IQR) of a total delay from the first onset of pulmonary illness (cough) to diagnosis (including patient and diagnostic delays) was 31 (19–48) days, ranged from 2 days to 212 days. Of the total delay longer than 21 days or 3 weeks, 209/309 (67.6%), the patients’ delay contributed 201/209 (96.2%). Patients, diagnostic, and total delay were lowest among antiretroviral treatment (ART) [Table 4].

### Factors associated with delays

In multivariable analysis, two variables were found statistical significance related to patient and total delays. Those who had a history of previous anti-TB treatment were 21% less likely to delay more than 15 days to be diagnosed when compared with those who had none (adjusted prevalence ratio = 0.79, 95% CI: 0.63–0.99). Those who had a history of weight loss were 12% times more likely to be delayed when compared with those who had not (adjusted prevalence ratio =1.12; 95% CI: 1.0–1.25) [Table 5].

## Discussion

We found that the median patients’ delay, diagnostic delay, and total delay were 30, 1, and 31 days, respectively. Patients’ delay was longer in duration among rural patients when compared with urban counterparts and those who visited hospitals when compared with those who visited health center. The diagnostic delay was longer among those who visited a health center or primary hospital that were found at rural setting when compared with those who visited at referral hospitals that found at urban and among those screened by symptom followed by chest X-ray (CXR) with cough <2 weeks duration compared with CXR group with cough ≥2 weeks and screened by symptom only.

We found 252 (81.6%) PTB patients who sought health care in ≥15 days, in which more than three out of four, 83.6%, were from rural. The median patients’ delay of our current study was longer when compared with studies done in Ethiopia, at a national level that reported 21 days,^[5]^ 17 days at Addis Ababa,^[18]^ and 25 days in southwestern Ethiopia^[24]^ but comparable with studies in the country that reported a median of patients’ delay 30 days at Adama, central Ethiopia,^[28]^ at Gamo zone, south Ethiopia,^[29]^ and Somali region, eastern Ethiopia.^[30]^ The difference might be due to the accessibility to health facilities, socioeconomic factors, and study populations. Besides, the patients’ delay was longer among rural residents. This could be related to the costs of transportation, accommodation, and others such as child care at home.^[28,30]^ Patients’ delay was low among ART patients. This could be due to the existing integrated program to control the TB/HIV co-infection that initiated the screening and diagnosis at an early stage.^[31]^

We found a very low diagnostic delay (2.6%) in the current study when compared with a study at the national level of Ethiopia that reported a median of 4 days,^[5]^ and a study from Addis Ababa reported 58.8% delayed more than 7 days.^[18]^ The diagnostic delay in the current study was lower than a study done in sub-Saharan Africa countries and other countries. In Uganda, median of 1 week among 44%,^[32]^ median of 1.7 weeks in Zambia,^[33]^ and at China diagnostic delay more than 2 weeks among 23.6%.^[34]^ Indeed, about a third (27.8%) patients were diagnosed in same-day in the current study that patients were very strongly favored in a study at Zambia.^[33]^ This very low diagnostic delay, as low as 2.6% with median of 1 day, in the current study might be related to the screening methods we used cough of any duration among people who sought health care for any reason, which was consistent with a study at Ethiopia,^[24]^ a study at Kenya,^[23]^ and the World Health Organization indicated as cough screening could reduce the delay^[17,24]^ but in our current study in addition to cough screening, among eligible it was followed by chest X-ray screening that is sensitive and can be detected TB before patients recognized the symptom.^[35]^

Further, screening by cough of any duration among people who sought health care reduced diagnosis delay. In this study, the diagnosis delay due to deficiencies of the health system such as lack of CXR, radiologist for CXR reading, and the longer diagnostic pathways was tried to be reduced through, for the health center, without CXR, patients were transported to a public facility with chest X-ray, the costs for transportation, imaging, and CXR interpretation by a radiologist were covered by the research project through agreement entered with the public facility. In Ethiopia, GeneXpert, AFB smear, and ant-TB drugs have been free but CXR has not free.^[21]^ As per NTP guideline, those with smear-negative or GeneXpert negative presumptive TB cases have been provided with antibiotics and appointed and re-checked after 2 weeks^[7]^ but many of the patients might not visit a health facility or select other health facilities that expose them to a longer delay.^[30]^

The majority of total delay was attributable to patients’ delay by reducing diagnostic delay. The median total delay of the current study was 31 days, which was lower than studies done in Ethiopia, at the national level, the median total delay was reported as 33 days^[5]^ and 50 days in the Somali region.^[30]^ It was also lower than studies from sub-Saharan Africa, a study from the Gambia reported 34 days^[36]^ and a study from Uganda reported a median of 13 weeks.^[32]^ In the total delay of the current study, the largest of delay (96.2%) was contributed by patients’ delay. This big contribution for the delay in PTB patients when compared with the health system or diagnostic delay was similarly reported in Ethiopia^[30]^ and in Uganda^[32]^ despite the contribution in our study was higher than other studies due to low diagnostic delay in the current study. This low contribution of diagnostic delay in the current study might be related to the screening for cough of any duration and/or followed by CXR screening we did among health care seekers.

Chest X-ray screening at the hospital with an expert (radiologist) reduced the diagnosis delay. For example, as Table 4 shows, median diagnosis delay at referral hospitals that have chest X-ray machines, GeneXpert, and radiologists was 1 day whereas it was 3 days at health center without CXR machine, radiologist, and GeneXpert; 2 days at the primary hospital that has CXR machine but had not staffed with a radiologist. This finding corroborated with the finding of systemic review and meta-analysis indicated as the use of CXR in reducing the diagnosis delay might vary by presence or absence of expert at the facility^[14]^ and the diagnosis delay was lower at a tertiary hospital in a study at Zambia.^[33]^ Patients visiting health center, reduced the patient’s delay as it has been accessible in the community but the diagnostic delay increased for the patients. This finding was similar to a study from the Somali region, Ethiopia^[30]^ and at the national level.^[5]^ This could be due to lack of appropriate diagnostic tools such as GeneXpert, CXR that aided the diagnosis of smear-positive and negative TB cases, and staffed with less required skills human powers^[21]^ despite many presumptive TB cases visited the health center at early when compared with hospital as we observed.

However, the current finding showed a longer diagnosis delay among the CXR group with cough <2 weeks when compared with their counterpart. The reason might be the waiting for the interpretation of CXR results, to be efficient with the use of GeneXpert for those who had cough <2 weeks, we performed the sputum laboratories only among those who had TB suggestive of CXR results by waiting for the results of CXR reading to perform sputum examination that could be increased the diagnostic delay among them. The algorithm <2 weeks cough duration followed by CXR screening reduced patients’ delay significantly, which is helpful to treat TB patients and revert the transmission. Screening for anyone regardless of cough duration reduced the delay in line with a study from Kenya.^[23]^

The strength of this study was executing screening by symptom and/or CXR by changing the usual screening of cough ≥2 weeks to cough of any duration for different target groups at wide-area coverage for both rural and urban settings at both hospitals and health center. However, the patients’ delay might be underestimated due to recall bias of the dates but we tried to control by asking the date at the start rather than the duration and giving appropriate time as they remembered. Besides, when CXR is used, the quality of CXR has to be considered to prevent the consequences of over diagnosis of TB that leads to treating patients without TB and/or under-diagnosis that leads to transmitting TB in the community. Errors and bias could be related to facility arrangements, type and technology of CXR machine used (conventional or digital), and turn-around time of imaging and reading.^[37–43]^ We exerted efforts to undue the challenges, particularly in meeting the quality of imaging and reading to meet the CXR preconditions outlined in a proclamation by the Ethiopian Government.^[44]^ We gave a thorough focus on CXR quality that images were taken by trained and qualified radiographers and read by senior radiologists. With all these considerations, the findings of this study can be evaluated further and implemented for facility-based screening and diagnosis of TB.

## Conclusion

Early screening using CXR minimized delays in the diagnosis of PTB among people with cough of any duration. Patients’ delay was largest and contributed great role in the delay of TB cases. Screening by cough of any duration and/or CXR among people seeking health care along with ensuring the availability of Xpert MTB/RIF assay and skilled human power at primary health care facilities are important to reduce patient and diagnostic delay of PTB in Ethiopia.

## Figures and Tables

**Table 1:** Sociodemographic characteristics of pulmonary tuberculosis among people sought health care at health facilities in Ethiopia

Characteristics	Frequency (%)
Gender	
Female	146 (47.3)
Male	163 (52.7)
Age groups	
0–14	34 (11)
15–24	81 (26.2)
25–34	97 (31.4)
35–44	51 (16.5)
45–54	29 (9.4)
≥55	17 (5.5)
Marital status	
Married	176 (57.0)
Single	109 (35.3)
Widowed	13 (4.2)
Divorced	4 (1.3)
Separated	7 (2.3)
Education level	
Never attended	147 (47.6)
Primary education	95 (30.7)
Secondary	40 (12.9)
education	
Tertiary education	12 (3.9)
Vocational	5 (1.6)
education	
Adult education	2 (0.7)
Religious education	8 (2.6)
Occupation	
Peasant	105 (34.0)
Housewife	59 (19.1)
Employed	31 (10.0)
Business	25 (8.1)
Student	49 (15.9)
None	28 (9.1)
Not applicable	12 (3.9)

**Table 2:** Patients’ delay of pulmonary tuberculosis among people screened by cough of any duration and/or chest X-ray

Characteristics	*n* (%)	Patient delay (days), median (IQR)	*P*
Delays	309	30 (16–44)	
Gender			
Female	146 (47.3)	30.5 (17–48)	0.3389
Male	163 (52.7)	30 (15–39)	
Residence			
Rural	226 (72.1)	31 (17–51)	0.0375
Urban	83 (26.9)	24 (15–34)	
HIV status			
Negative	72 (23.3)	30 (15–43.5)	0.5844
Unknown	226 (73.1)	31 (17–48)	
Positive	11 (3.6)	29 (16–31)	
Attended health facilities			
Hospitals	274 (88.7)	31 (17–51)	0.0037
Health center	35 (11.3)	20 (10–31)	
Types of PTB			
Bacteriological confirmed	185 (62.7)	31 (17–51)	0.0642
Bacteriological/smear negative	110 (37.3)	30 (15–35)	
Previous TB treatment			
Yes	38 (12.3)	18.5 (9–32)	0.0148
No	270 (87.7)	31 (17–46)	
Visited facilities for cough care			
Yes	300 (97.0)	31 (17–45)	0.0771
No	9 (3.0)	15 (14–24)	
First visited health facilities			
Public/missionary health facilities	284 (92)	30.5 (16–43.5)	0.6756
Private health facilities	21 (6.7)	30 (16–56)	
Drug store	3 (1)	53 (29–64)	
Other	1 (0.3)	-	

IQR: Interquartile range, CXR: Chest X-ray, TB: Tuberculosis, PTB: Pulmonary TB, HIV: Human immunodeficiency virus

**Table 3:** Diagnostic delay of pulmonary tuberculosis among people screened by cough of any duration and/or chest X-ray

Characteristics	*n* (%)	Diagnostic delay (day), median (IQR)	*P*
Delays	309	1 (0–3)	
Gender			
Female	146 (47.3)	1 (0–2)	0.7105
Male	163 (52.7)	1 (0–3)	
Residence			
Rural	226 (72.1)	1 (0–3)	0.6018
Urban	83 (26.9)	1 (0–3)	
Facility location			
Urban	168 (54.4)	1 (0–2)	<0.0001
Rural	141 (45.6)	2 (1–4)	
Algorithms			
Non CXR group	14 (4.5%)	0 (0–1)	0.0221
CXR group with <2 weeks cough	44 (14.2)	2 (1–3.5)	
CXR group with ≥2 weeks cough	251 (81.2)	1 (0–2.5)	
Sputum test results			
Positive	185 (62.7)	1 (0–2)	0.0423
Negative	110 (37.3)	2 (1–3)	
Previous TB treatment			
Yes	38 (12.3)	1.5 (0–3)	0.8404
No	270 (87.7)	1 (0–3)	
Diagnosed at facilities had CXR			
Yes	247 (88.7)	1 (0–2)	0.0001
No	35 (11.3)	3 (2–4)	
Diagnosed at facilities had radiologist			
Yes	168 (54.4)	1 (0–2)	<0.0001
No	141 (45.6)	2 (1–4)	
Diagnosed at facilities had GeneXpert			
Yes	247 (88.7)	1 (0–2)	0.0001
No	35 (11.3)	3 (2–4)	

IQR: Interquartile range, CXR: Chest X-ray, TB: Tuberculosis, PTB: Pulmonary tuberculosis

**Table 4:** Comparing delays of pulmonary tuberculosis among people screened by cough of any duration and/or chest X-ray

Facilities, screening algorithms, and units	Patients delay	*P*	Diagnostic delay	*P*	Total delay	*P*
Types of health facilities						
Referral hospitals with CXR, GeneXpert, smear microscopy, and radiologist at urban setting	30 (17–45.5)	0.0102	1 (0–2)	0.0001	31 (18.5–48.5)	0.0243
Primary hospital with CXR, smear microscopy, and GeneXpert at rural	31 (17–51)		2 (1–4)		34.5 (19–52)	
Health center only with smear microscopy at rural	20 (10–32)		3 (2–4)		22 (14–34)	
Screening algorithms						
Non CXR group	30 (19–31)	0.0001	0 (0–1)	0.0221	30 (20–32)	0.0001
CXR group <2 weeks cough	7 (6–10)		2 (1–3.5)		10 (7.5–12)	
CXR group ≥2 weeks cough	31 (22–56.5)		1 (0–2.5)		33 (24–58.5)	
Departments/units						
OPD and RCH	31 (16–45.5)	0.5594	1 (0–3)	0.0409	31 (18–50.5)	0.4728
Anti-retroviral patients	27 (16–31)		0 (0–1)		27.5 (19–31)	
ANC and PMTCT	31 (30–32)		1 (0–2)		32 (30–34)	

CXR: Chest X-ray, TB: Tuberculosis, PTB pulmonary TB, OPD: outpatients department, RCH: Reproductive and child health, ANC: anti-natal care, PMCT: Prevention from mother to child transmission

**Table 5:** Factors associated to patients’ delay and total delay among pulmonary tuberculosis, Ethiopia

Characteristics	Patient delay			Total delay		
	*n*	cPR with 95% CI	aPR with 95% CI	*n*	cPR with 95% CI	aPR with 95% CI
Total	252			209		
Gender						
Female	124	1	1	100	1	1
Male	128	0.92 (0.83–1.02)	0.92 (0.83–1.02)	109	0.97 (0.83–1.13)	0.96 (0.82–1.12)
Location						
Rural	189	1.1 (0.96–1.26)	1.05 (0.92–1.17)	161	1.05 (0.00–1.5)	1.16 (0.95–1.41)
Urban	63	1	1	48	1	1
Sputum test results						
Positive	157	1.08 (0.96–1.21)	1.04 (0.92–1.17)	131	1.12 (0.95–1.34)	1.07 (0.90–1.28)
Negative	86	1	1	69	1	1
Previous TB treatment						
Yes	25	0.78 (0.62–0.99)	0.79 (0.63–0.99)	18	0.67 (0.47–0.95)	0.70 (0.50–0.97)
No	226	1	1	190	1	1
History of weight loss						
Yes	83	1.11 (1.01–1.23)	1.12 (1.0–1.25)	72	1.20 (1.02–1.39)	1.22 (1.04–1.44)
No	169	1	1	137	1	1

TB: Tuberculosis, PTB pulmonary TB, cPR: Crude prevalence ratio, aPR: Adjusted prevalence ratio, CI: Confidence interval

## Data Availability

All important data used for this article included in the main text. Datasets for the article are available upon reasonable request from the corresponding author.
